# Bio-Inspired Knee Joint: Trends in the Hardware Systems Development

**DOI:** 10.3389/frobt.2021.613574

**Published:** 2021-09-01

**Authors:** Appolinaire C. Etoundi, Chathura L. Semasinghe, Subham Agrawal, Alexander Dobner, Aghil Jafari

**Affiliations:** Bristol Robotics Laboratory, University of the West of England, Bristol, United Kingdom

**Keywords:** bio-inspired mechanism, prosthesis, robotic device, knee joint, control strategy

## Abstract

The knee joint is a complex structure that plays a significant role in the human lower limb for locomotion activities in daily living. However, we are still not quite there yet where we can replicate the functions of the knee bones and the attached ligaments to a significant degree of success. This paper presents the current trend in the development of knee joints based on bio-inspiration concepts and modern bio-inspired knee joints in the research field of prostheses, power-assist suits and mobile robots. The paper also reviews the existing literature to describe major turning points during the development of hardware and control systems associated with bio-inspired knee joints. The anatomy and biomechanics of the knee joint are initially presented. Then the latest bio-inspired knee joints developed within the last 10 years are briefly reviewed based on bone structure, muscle and ligament structure and control strategies. A leg exoskeleton is then introduced for enhancing the functionality of the human lower limb that lacks muscle power. The design consideration, novelty of the design and the working principle of the proposed knee joint are summarized. Furthermore, the simulation results and experimental results are also presented and analyzed. Finally, the paper concludes with design difficulties, design considerations and future directions on bio-inspired knee joint design. The aim of this paper is to be a starting point for researchers keen on understanding the developments throughout the years in the field of bio-inspired knee joints.

## 1 Introduction

The knee joint is one of the largest joints with a complex structure in the human body and performs a crucial role in many activities of daily living (ADLs) such as standing, walking, running and climbing stairs. Given the complexity of biomechanics and structure of the lower extremity, it is extremely hard to replace a missing lower limb in the human body with a transfemoral (above knee) prosthesis. The prosthetic knee joint plays a significant role in the transfemoral prosthesis during the gait cycle due to the fact that it articulates two bodies from the hip side and the ankle side (Convery, 1981). Human gait analysis is crucial in designing a lower limb prosthesis and the human gait can be divided to two phases; stance and swing (Agostini et al., 2014). Stance represents the entire phase which the foot contacts with ground and swing phase is the period which foot is in the air during the limb advancement (Agostini et al., 2014). There are passive prosthetic knee joints that are used in transfemoral prostheses to support walking by restricting knee flexion during stance phase and releasing the flexion at swing phase (Dabiri et al., 2010). During the swing phase, the prosthetic knee flexion and extension are controlled by the hydraulic or spring loaded dampers. The active prosthetic knee joints are incorporated with sensors and a microprocessor for controlling the actuators to obtain the joint movements by analyzing the data gathered from user intention and the environment (Kahle et al., 2008; Dabiri et al., 2010). There are also semi-active prosthetic knee joints which are adopted with actuators used only for changing the damping behavior depending on variations such as locomotion condition, walking speed, amputee’s weight and external environmental impacts (Kahle et al., 2008). In the power-assist wearable devices like HAL by Cyberdyne Inc.^1^ or MuscleSuits devices by Innophys Co. Ltd,^2^ externally powered artificial knee joints are adopted to achieve mobility and load bearing by paraplegics. The knee joint movements in most of these devices are controlled by electro-magnetic motors while a few use pneumatic or hydraulic actuators which bring high force/weight ratio. However, in most prostheses and power suits, it has been impossible to achieve the natural movement of the biological knee due to the key fact that kinematic and spatiotemporal asymmetries between the artificial and biological knee joint during flexion-extension and movements of the knee joint in a single plane (Author Anonymous).

The human body is built via evolution to achieve highly optimized joint movements with low power consumption. Therefore, bio-inspiration design has been one of the most prominent aspects to be considered in the research field of prostheses, power-assist suits and mobile robots (Liu et al., 2016). This benefits the amputees and aged society by eliminating most of the limitations found in their prosthetic devices and power-assist suits. The knee joint structure has a significant effect in terms of size, weight, stiffness, inertia, power demand, aesthetic appearance, durability and shock absorbence towards the functional performance and user acceptance of the lower limb prosthesis or exoskeleton (Liu et al., 2016). Therefore, a broad study on the anatomy and biomechanics of the human knee joint is important to identify the fundamental requirements for designing and controlling an ideal artificial knee joint. A well-designed robotic knee will enable the prosthetic leg or the exoskeleton to be functioned with little energy dissipation and to passively accommodate disturbances from dynamic environment with a minimal need from a controller to sense and response them. In the current research field, for controlling the robot joints there can be seen many advanced control strategies such as improved recurrent neural network-based manipulator control (Su et al., 2020a), extended electromyography (EMG) control with deep vision learning (Su et al., 2020b), and control with adaptive compensator (Su et al., 2019). However, even the best robot control cannot extract efficient human-like performance from a prosthetic leg/exoskeleton when the leg dynamics require unreasonably high-bandwidth controllers to maintain the stability and agility against the impacts and other perturbations from external environments and terrain irregularities.

The latest technological enhancements may enable deriving an ideal replacement for the human knee joint. However, it is necessary to have a better perspective on the design characteristics and functionality of existing bio-inspired knee joints for developing an ideal knee joint. In that context, this paper presents the state-of-art bio-inspired knee joints found in prostheses and power assist suits during the last decade. The hardware system of research level bio-inspired knee joints are reviewed in this paper. In this review we highlighted the design considerations for an ideal artificial knee joint based on the biological structure, summarize the major works on bio-inspired knee joints to date and identify the research gaps and knowledge paths for future researchers. For a systematic literature review, research publications from different databases like IEEExplore, Google Scholar and Google Patents are retrieved using a search strategy.

## 2 Anatomy and Biomechanics of Human Knee Joint

**FIGURE 1:**
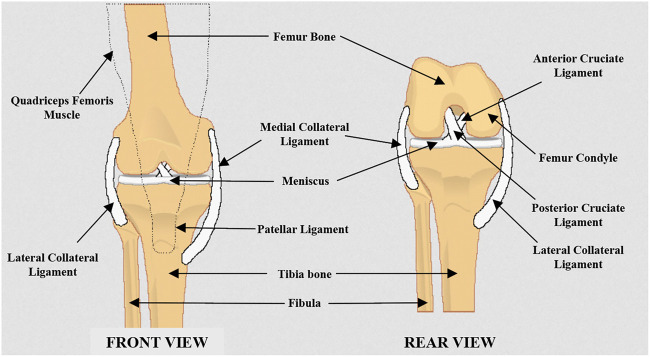
Anatomy of human knee joint (front view and rear view).

Structure of the human knee joint is shown in Figure 1. In the design of bone’s structure, the femur has a convex surface in the femur condyle where it contacts with a matching concave surface of the tibia bone. The meniscus provides a cushion between the contact surface of tibia and femur while absorbing the impact forces on the knee joint and providing stability (Convery, 1981). The fibula is the long and lateral bone in the lower limb which stays parallel to the tibia bone. It does not attach to the knee joint. However, it plays a major role by being an anchor point to many muscles and ligaments while stabilizing the ankle joint (Gupton and Terreberry, 2018). The quadriceps femoris, is a group of four muscles which supports the knee joint extension by muscle contraction. The hamstrings muscles group supports the knee flexion while extending the hip (Gupton and Terreberry, 2018). Two collateral ligaments are attached on the fibula and tibia in both lateral and medial sides (see Figure 1). These two ligaments get tight when the knee is extended and become less tight at joint flexion while keeping the femur and tibia bones together by preventing any relative lateral or medial movement (Gupton and Terreberry, 2018). However, this ligament structure is not adequate for a stable anteroposterior motion at the joint (Sakai et al., 2015). Therefore, anterior and posterior cruciate ligaments (ACL and PCL) are found within the knee joint which connect the femur and tibia while crossing each other to stabilize the anteroposterior motion (Sakai et al., 2015). This ligament structure brings a crossed four-bar mechanism with moving instantaneous center of rotation (ICR) found at the cross point of ACL and PCL that follows in an elliptical path (Fu et al., 1993).

**FIGURE 2:**
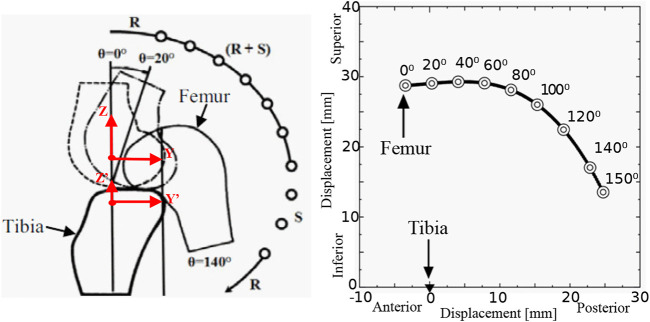
Biomechanics of human knee joint (Fu et al., 1993; Hinson Marilyn, 1981); displacement of femur with respect to the flexion angle of the knee.

The knee joint flexion/extension motion is a combination of rolling and sliding without having a fixed center of rotation. In Figure 2, the rotation of femur’s origin in sagittal plane is shown with respect to the tibia’s origin on a defined coordinate system. In the coordinate frame, the vertical axis represents the superior-inferior movement of rotational axis and horizontal axis represents the anteroposterior movement of the rotational axis (Hinson Marilyn, 1981). Generally, a healthy adult knee joint has a range of motion from 0 to 150° where 0° is at fully extended and 150° is at fully flexed position (Hinson Marilyn, 1981). According to Figure 2, on a healthy adult, the rotational axis of the joint moves approximately 30 mm in anteroposterior direction and 15 mm in superior-inferior direction. However, there are no common trends on movements in other directions within frontal or horizontal planes (Hinson Marilyn, 1981). The fact that the knee joint gets self-locked at the fully extended position is another key aspect in the human knee joint. This feature reduces the energy demand from quadriceps femoris muscle at the standing position (Fu et al., 1993). It is identified that the torque requirement for knee joint movement does not depend on the gender but on the weight and height of the body (Kerrigan et al., 2000). During human walking, the hamstrings muscles group generates peak flexor torque of 0.34±0.15 Nm per kg-m which is normalized to weight and height (Kerrigan et al., 2000). On an average healthy adult, approximately 57.8 Nm of maximum torque is needed from the quadriceps femoris muscle when standing up and it is constantly needed during the joint angle range of 80–150° (Masahiro et al., 1990).

## 3 Review on Modern Bio-Inspired Knee Joints

Modern bio-inspired knee joint founds within the last decade are briefly reviewed in this chapter. After screening the abstract, description and content of publications, 12 research works were selected based on their salient technological features and the availability of information on design or control or both for the review.

### 3.1 Trend in Bio-Inspired Knee Joints

With the rapid development within the robotics and autonomous systems (RAS), the many solutions are introduced for transfemoral amputees in both research and commercial level to achieve the user needs in different conditions. The modular based transfemoral prostheses came into the prosthetic market to make the amputee more effective and comfortable on their activities on locomotion. These devices provide the opportunity in replacing (limb transition) the leg prosthetic unit with or without the knee joint for the given specific tasks. Limb transition now has become a key concern for the transfemoral amputee for better body balance maintenance and comfort during locomotion.

However, the limb transition makes the amputee feeling uncomfortable in ADLs as s/he must change the leg to achieve specific locomotion like running, walking or cycling. As an example, amputee who uses a leg prosthetic for general walking is not able to use the same prosthetic device for running or cycling. So, the amputee has to carry different prosthetic legs in a journey to achieve the different locomotion patterns. When considering the prosthetic leg for different locomotion patterns, they are built for achieving the recommended shock absorbance, desired stiffness, required gait pattern, robustness and reliability. The knee joint embedded within these leg prostheses play a significant role on the mechanical performance of the artificial limb in terms of stability, inertia, stiffness and load bearing. If a knee joint can be developed to handle all these significant characteristics to perform ADLs in human like, safe and efficient manner, then the limb transition will not be needed for the transfemoral amputees to achieve different locomotion patterns. Therefore, a bio-inspired knee joint may enable to make the amputee’s life more comfortable and to achieve the human like motion as the biological knee is developed with the remarkable characteristics embedded within the joint to achieve different locomotion patterns.

### 3.2 Design of Research Level Bio-Inspired Knee Joints

In the musculoskeletal system, the shape, flexibility and structural compliance are key features for the adaptation to external forces from dynamic environment and internal stress distribution which maintains the structural integrity. These features also support natural movement of lower limbs with low energy consumption and strong locomotion capabilities over rough terrains. In the recent researches on lower limb prostheses and exoskeleton, the designs of bio-inspired knee joints have notable features on three main aspects; bone structure, muscles and ligaments structure and actuation method.

**FIGURE 3:**
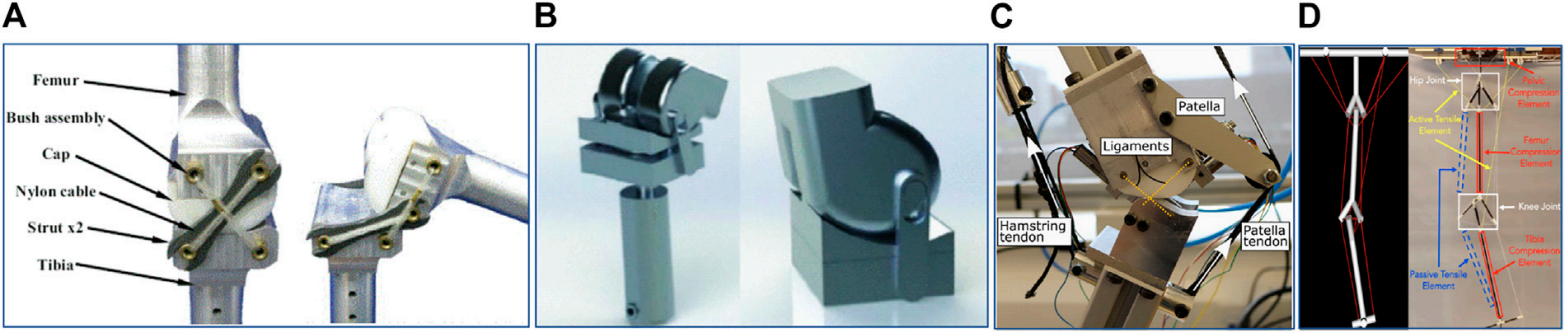
Bio-inspired knee joint based on bone structure. **(A)** Condylar Hinge Joint by Etoundi et al. (2011) **(B)** Knee joint for bipedal robot by Steele et al. (2017) **(C)** Biomimicking design for mechanical knee by Russell et al. (2018) **(D)** Tensegrity flexural joints by Jung et al. (2018).

Considering the bone structure, a research was published in 2011 where a condylar hinge joint is designed (see Figure 3A) for both prostheses and mobile robots (Etoundi et al., 2011). The contact surfaces of both tibial and femoral component are designed based on the curved profile which mimics the condylar surfaces found in the human knee to achieve the good strength on the knee structure, high conformity and stiffness at joint movements (Etoundi et al., 2011). This condylar hinge joint provides a significant mechanical advantage over a pin-jointed hinge from large range of motion in given volume, better impact force distribution and increased life performance of the joint. In 2017, Steele et al., proposed a new design concept for condylar hinge joints for the knee joint with consideration of dynamic pressure changes at contact surfaces which causes wear and poor stability at joint flexion (Steele et al., 2017). They use a pneumatic piston (see Figure 3B) attached in the tibial component to dynamically control the pressure found in the contact surface between the tibial and femoral component. This brings additional control, stability and stiffness to the joint at its flexion. It also minimizes the slip on the contact surfaces during rolling and sliding at joint flexion/extension which causes friction and wear. In their design they have also considered the function of patella found in the human knee joint (Steele et al., 2017). It extends the moment of arm of the knee joint during extension while keeping the joint compact and lightweight. This concept of design makes a knee joint with less power consumption, a high range of motion, less wear and improved stability compared to a conventional hinge joint of same dimensions. With the concern of condylar shape of bones at the contact surfaces, another bio-mimicking design (Russell et al., 2018) is proposed for a mechanical knee joint in 2018. As shown in Figure 3C, a patella structure is embedded in the condylar joint which provides similar moment arm profile found within the human knee. From the experiments conducted on patella arrangements, they have found that the patella fixed on the tibial component with a rigid bracket provides better fit to human knee moment arm (Russell et al., 2018). This bone structure also reduces the risk that may arise from hyper-extension of the knee joint or sudden deceleration during knee extension at the fully extended stage. In addition, this arrangement benefits in less power demand for actuating knee extension which is significant in ADLs such as sit-to-stand and walking stairs (stair ascent) activities.

The biological musculoskeletal system is built with structural compliance and flexibility to accommodate external forces and better internal stress distribution (Jung et al., 2018). In 2018, a research team from the United States proposed a tensegrity structure which can be adopted in augmenting human gait, specially in rehabilitation (Jung et al., 2018). The lower limb of this structure which consists of pelvis, femur and tibia is made from compression elements and they connected each in series while suspended within a network of cables (see Figure 3D). Carbon fiber rods are used as the compression elements. As shown in the labelled diagram in Figure 3D, the attachment point of the tibial component at the knee joint is designed as a Y-shape and the femoral component follows a three-rod base shape. Both links can be bonded together by composing a flexural knee joint. The proof of concept of this design has shown human like knee movements and ability for handling unanticipated impedance. However, they are still working on the design concept to be used in patient’s rehabilitation. The key advantage of such a design is the ability to use a knee joint with better adaptability in unpredictable locomotion conditions such as uneven terrain.

**FIGURE 4:**
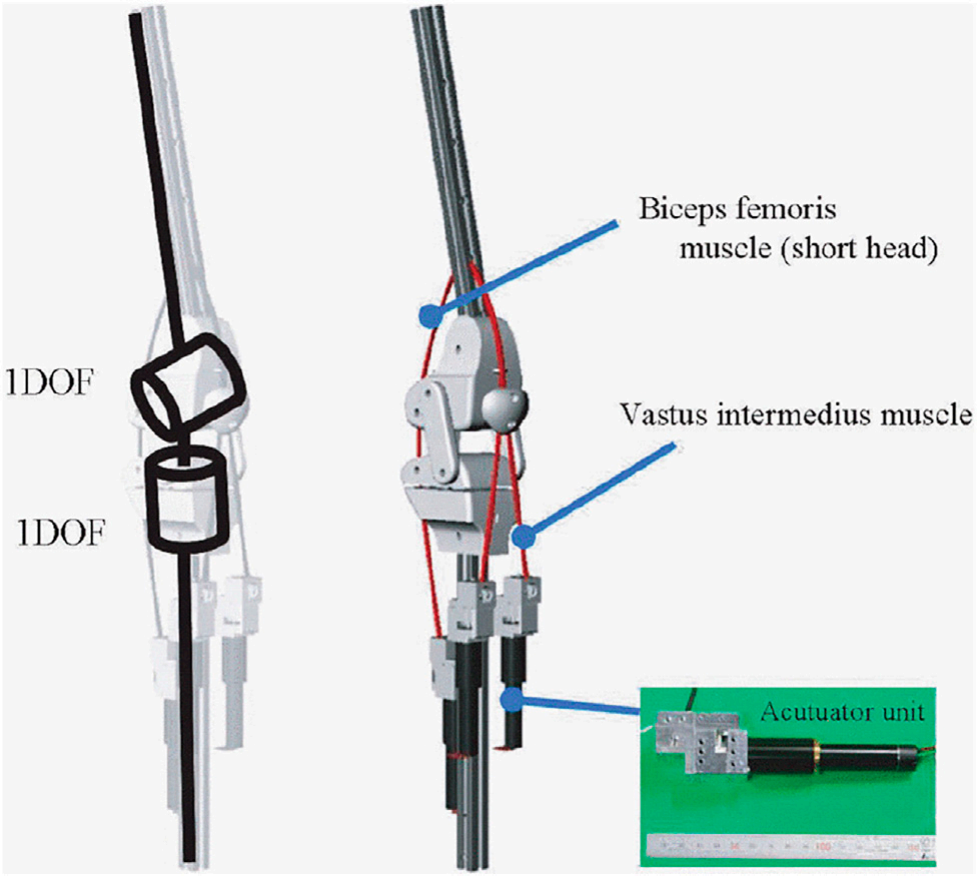
3D model of a bio-inspired knee joint with a patella structure (Asano et al., 2011).

Asano et al. has conducted a research (Asano et al., 2011) on novel musculoskeletal humanoid knee joint to achieve three key functions in human knee joint; rolling and sliding, patella movement and screw-home mechanism. In this knee structure, there is a mechanism for sliding the patella on a groove along with the femoral component (Asano et al., 2011). A tendon which acts as quadriceps muscles is attached between femoral and tibial component through the patella, (Asano et al., 2011). From the experimental results, it has proved that the patella structure (see Figure 4) causes lower power requirement during the knee extension by increasing the moment arm (Asano et al., 2011). The human knee joint has a rotational function over yaw axis which enable various foot directions when the joint is flexed (Asano et al., 2011). However when it fully extends, this function is disabled for bringing stability during stance and upright position. This behaviour is known as the screw-home mechanism which self locks the joint at complete extension (Asano et al., 2011). In the novel musculoskeletal humanoid knee joint design, this yaw axis DoF is implemented by separating the tibial component into two sub-components and connecting them via a metallic rod. A groove is designed in the lower tibial part to restrict rod movement in fully extended knee and it disengages from the groove when joint flexes. (Asano et al., 2011). The bone structure of this knee joint brings energy efficiency, human like movements, lightweight and compactness. However, this design brings adverse results on strength, load bearing, durability and reliability due its design complex.

**FIGURE 5:**
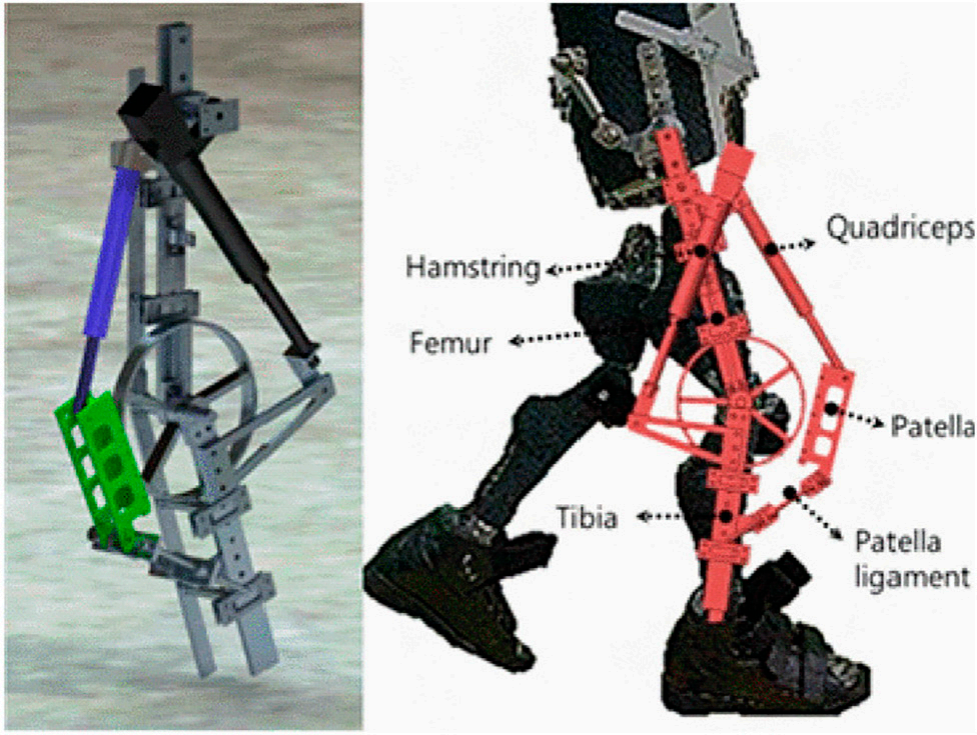
Knee joint actuation mechanism for gait rehabilitation exoskeleton (Pyo et al., 2010).

**FIGURE 6:**
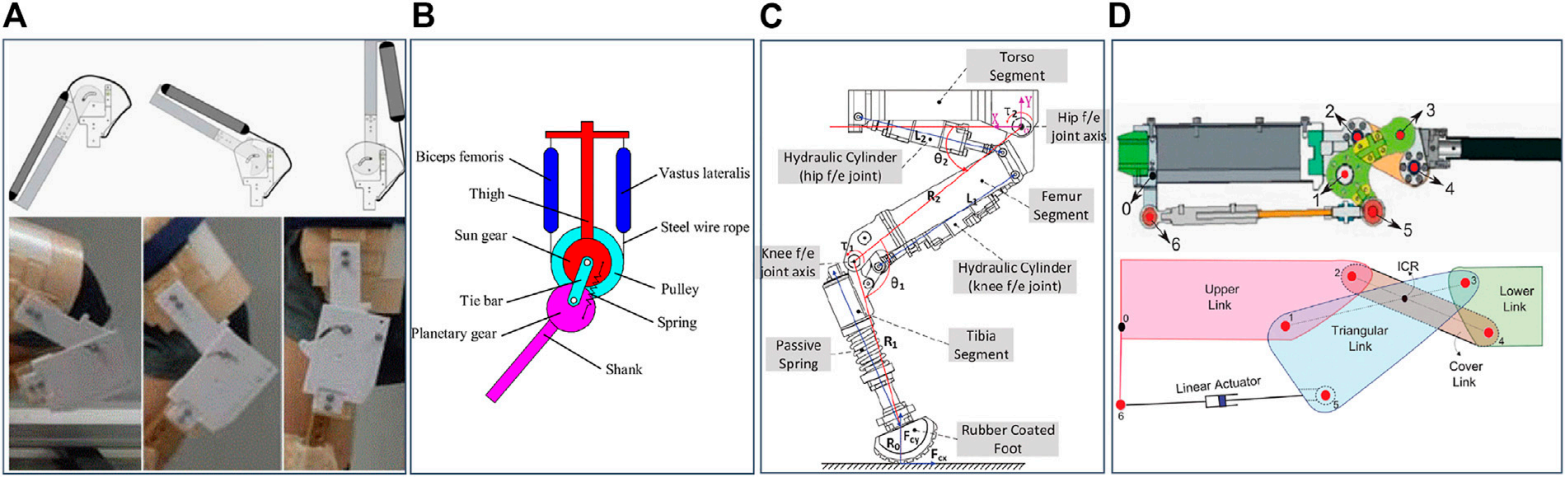
Bio-inspired knee joint based on muscle and ligament structure. **(A)** Knee joint for power assist suit by Sakai et al. (2015) **(B)** Knee joint for biped robots by Liu et al. (2016) **(C)** Articulated robotic leg by Li et al. (2017) **(D)** Knee joint for quadruped robot by Khan et al. (2015).

To mimic the natural movements of human lower limb, it is important to study the bio-mechanical structure of the human leg built with a muscle and ligament arrangement. Based on human biomechanics, a conceptual design was proposed in 2010 for an active knee orthosis which supports gait cycle and leg movements of patients with lack of muscle force (Pyo et al., 2010). Two linear actuators are adopted in the design to substitute the muscle forces (see Figure 5). One actuator does the flexion function and other does the extension where they mimic the functions of the hamstrings and quadriceps muscles group (Pyo et al., 2010). The desired knee joint angle is obtained by controlling these two actuators and the hardware design of the joint improves the competence over a simple revolute joint. In this proposed system, the gait cycle is achieved efficiently by distributing high velocity and high power requirements for both kinematic chains during each phase of leg movement. Therefore this design brings efficient power consumption that increases the functional lifetime of the device. Sakai et al. proposes a wire-pulley mechanism for bio-inspired knee joint to be used in power assisted suits (Sakai et al., 2015). The relative motion between components attached on femoral and tibial bodies is achieved by a wire rope that is connected to a pneumatic artificial muscle (PAM) actuator (see Figure 6A). The actuation of PAM varies the joint angle in a non-circular sliding path that incorporates the anteroposterior motion found in the human knee (Sakai et al., 2015). This design concept allows a higher flexion angle and avoids the possibility of dangerous over-extension.

The knee joint should be compliant to prevent any damage on the joint structure due to large shocks (Liu et al., 2016). The elasticity of PAMs can provide the spring-like compliance behavior to the joint (Liu et al., 2016). In 2016, a human-like knee joint was proposed that uses a planetary gear transmission mechanism and PAMs. In this planetary gear structure, the sun gear is attached on the femoral component and planetary gear on the tibial component as shown in Figure 6B. Two PAMs were used in the design to achieve the flexion/extension of the joint. One end of the PAMs are connected to femoral component using wire ropes and the other ends are connected in series from a steel wire rope which winds on the pulley. The pulley is attached in a certain way, so as to move the planetary gear around the sun gear. By these, the joint flexion/extension is achieved by the pulley rotation which is driven by PAMs. An extension spring is inserted in the joint to support knee extension driven by the spring load that brings natural leg swing during the walking. However the knee extension can also be controlled using PAMs where necessary in the locomotion (Liu et al., 2016). This design is inspired by a bio-mechanical structure, thus achieving better joint compliance and human-like knee movement. In addition, this gives larger range of motion of knee rotation and low power requirements for operation.

Stiffness of the lower leg needs to be changed for adaptation on different terrain during locomotion. Li et al. proposes a bio-inspired lower limb which uses spring dampers and hydraulic actuators for dynamic behavior (Li et al., 2017). The knee flexion/extension movement is achieved by a hydraulic actuator which mimics the functionality of both hamstrings and quadriceps muscle groups (see Figure 6C). However the knee joint design is limited to a simple hinge joint which has fixed rotational axis. In the control architecture, the hydraulic actuator plays a significant role in achieving compliance control over impact loads and shock absorbence which is observed in the biological muscle and ligaments structure (Li et al., 2017). It is also possible to achieve the compliance using spring dampers attached in series or parallel with the electrical actuators. However, it is challenging to build compact and lightweight joint due to the components needed for achieving compliance compared to a hydraulic design.

Ligaments support a smooth and stable motion in the knee using an inverted parallelogram mechanism which has a changeable ICR at different joint angles (Etoundi et al., 2013a). In the research based on condylar hinge joint by Etoundi et al. (2011, 2013a), this ligament structure is implemented to achieve human-like motion at knee movement and also it fits best with the cam profile of a condylar shaped bone structure. However, ACL and PCL are placed on each sides of the joint in their design instead of locating them in the middle where they are found in the human knee (Etoundi et al., 2013a). This arrangement retains the stability of the joint and alleviates the need of additional ligaments that prevents medial and lateral displacement between tibial and femoral components. In addition a continuous cable is used in this design to achieve the arrangement and functionality of both ACL and PCL as shown in Figure 3A. This concept avoids any complex holes structure or cut-outs in condylar surfaces of the joint. With this ligament arrangement, rolling and sliding function in human knee has been brought to the joint, which results in human-like movement and higher range of motion.

In the development of a hydraulic quadruped robot, a bio-inspired knee joint mechanism is proposed to achieve strong and fast locomotion in rough terrain (Khan et al., 2015). The knee joint is designed based on the ligament structure found in human knee. The design achieves changeable ICR which is a primary factor for human like knee motion. The joint consists of two main links: which are in triangular and rectangular shape that connect the femoral and tibial attachment links. As shown in Figure 6D, the rectangular shaped link is attached on both femoral and tibial links with its nodes at both ends. In the triangular shape link, two nodes are attached to the tibial and femoral components and the third node is directly connected to the linear actuator which drives the knee joint rotation with changeable ICR. This design provides compact and lightweight structure that brings mechanical advantage over a simple revolute joint operated by a linear actuator. In this research (Khan et al., 2015), objective functions and kinematic models are considered to achieve optimal design variables for links. According to the experimental results, the design exhibits larger motion range and efficient torque profile during the knee flexion/extension in a dynamic environment.

**FIGURE 7:**
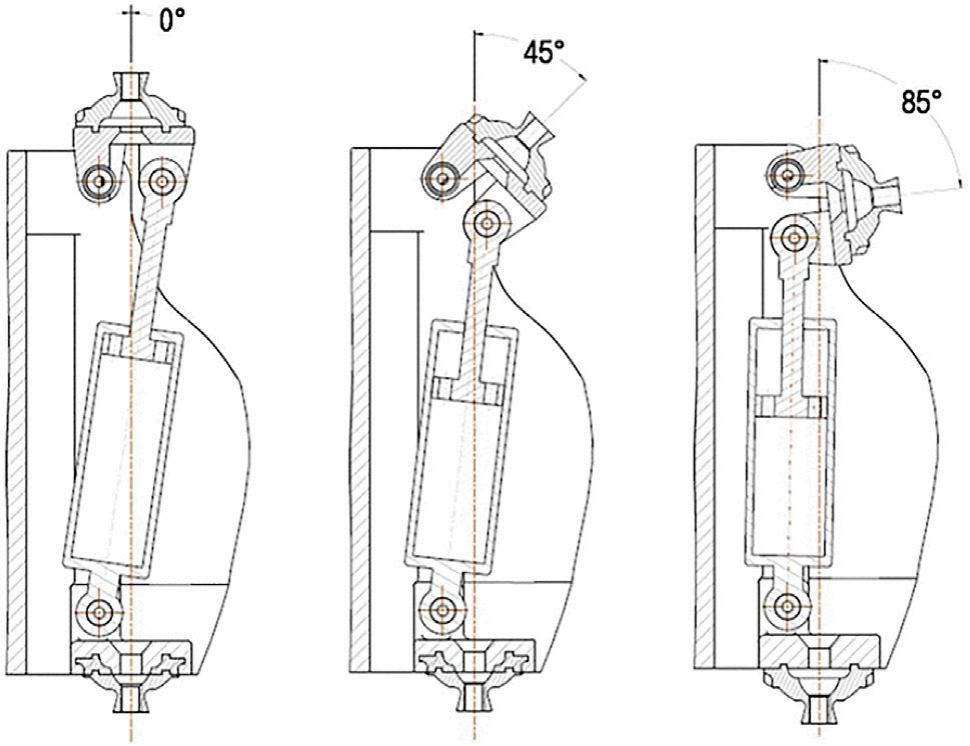
Mechanical design of prosthetic knee joint with magnetorheological actuator (Torrealba et al., 2010).

Considering the actuation methods for bio-inspired knee joints, some researchers have considered hydraulic actuators (Li et al., 2017; Khan et al., 2015) and some designs are based on pneumatic actuators (Liu et al., 2016; Sakai et al., 2015). These pneumatic and hydraulic actuation methods bring advantages such as high power to weight ratio, high compliance and follow the load curves of biological muscle functionality. However, these actuation methods adversely affect mobility, aesthetic appearance, weight and size when considering the overall system. For example, although the knee joint device incorporates all the bio-inspiration features, bulky and heavy additional components such as compressors and pumps are needed for generating pneumatic or hydraulic power. In research of developing a biomechatronic knee prosthesis for transfemoral amputees, a magnetorheological actuator is used to control the knee joint which follows a monocentric design (see Figure 7) (Torrealba et al., 2010). This actuator controls the resistance over the piston movement using the current passing through an electromagnetic structure found inside the device. Therefore the knee joint rotation is controlled according to the current value that is provided to the actuator. The knee is flexed passively according to the torque applied externally and controlled the knee flexion angle using electric current which generates a resistive force on knee flexion (Torrealba et al., 2010). However, power for this actuation method is from a source such as batteries. By embedding bio-mechanical energy harvesting methods, the need for battery charging by external sources or replacement of batteries might be eliminated.

### 3.3 Control Strategies of Research Level Bio-Inspired Knee Joints

The control strategies of the general knee prostheses can be classified into two categories; active and passive. In most commercial knee joint products, passive control systems are identified which use springs, hydraulic dampers and mechanical locks. Though some passive systems are able to generate harmonious gait patterns during different speeds of walking, they are not capable for multi-tasks that are found within ADLs. For instance, the same knee joint cannot be used for walking and running. With the technological enhancements, active knee prostheses are being developed that obtain human-like lower limb movements with adaptation towards the dynamic environment. At present the most widely used control strategy in active knee prostheses is finite-state control combined with variable impedance control (Gregg and Sensinger, 2013; Lawson et al., 2014). The variable impedance control is based on the sensory information of stiffness of the spring, and on the damping coefficient that depends on gait phase and virtual impedance so as to follow the human-like locomotion behaviour (Gregg and Sensinger, 2013). Though this control strategy provides fair human gait in locomotion, it has many limitations compared to biological motor control strategy (Torrealba et al., 2010). This finite-state architecture limits the adaptive capability as the knee prosthesis is controlled by states rather than being continuous (Torrealba et al., 2010). In addition, it is hard to obtain physiological impedance values that are required for impedance modulation. So the parameters for knee joint movement have to be manually tuned. This process is a tedious and time consuming (Aghasadeghi et al., 2013). As an alternative to this strategy, Pfeifer et al. (2012) propose a model-based approach which obtains values from joint stiffness estimation for visco-elastic impedance control. However this approach also has the limitation of poor adaptability towards the dynamic environment. Due to all these limitations, researchers moved to develop bio-inspired control strategies that brings human-like movement into the dynamic environment with adaptation.

One approach proposes a bio-inspired impedance controller that brings physiological modulation of joint dynamics such as angular velocity, angular moment and joint angle during locomotion (Pagel et al., 2017). With the inspiration from intrinsic and reflexive pathways found in the human motor control architecture, a neuro-musculoskeletal impedance model is implemented for achieving the physiological modulation. Based on the principles of optimality in both human motor control and physiology, human model reference adaptive controller is developed for optimal adaptation in knee joint angle and moment towards the dynamic environment during locomotion (Pagel et al., 2017). A pilot study is conducted with three participants to test this control strategy and the results have shown better natural behavior and adaption in locomotion. However this strategy still needs improvements in the calculation of reference trajectories in human gait and enhancement in dynamic human gait control, which is non-linear and time-variant.

Control via a central pattern generator (CPG) is another intelligent control strategy that provides more natural control over lower limb (Marder and Calabrese, 1996). This strategy generates the movement coordinates for lower limb motion during the human gait. These coordinated movements are achieved from rhythmic patterned outputs that are generated through a neural network driven on a mathematical model coupled with differential equations (Marder and Calabrese, 1996). In general, this control strategy is used with an oscillator model such as amplitude-control phase oscillator in Marder and Calabrese (1996) and neural oscillator in Schrade et al. (2017). The model is linked with a CPG via neurons in a neural architecture and it tracks the phases in gait cycle for producing rhythmic oscillatory output. This output contains information about flexion and extension of the knee joint and the CPG uses this information to control the joint with respect to the other leg movement.

### 3.4 Design Consideration for a Bio-Inspired Robotic Knee Joint Development

After reviewing the hardware design and control strategies of recent bio-inspired knee joints developments, it is perceptible that the researchers have concerned on several design attributes to bring bio-inspiration to the robotic knee joints. In the hardware design, the bio-inspiration is brought to the joint by mainly focusing on three bio structures namely, bone, ligament and muscle. Joint design by mimicking these three structures has enabled the robotic knee joint to perform the human-like knee movements with joint compliance, flexibility and high robustness in the dynamic environment. As other design attributes, researchers have considered the knee joint characteristics such as range of motion, functional movements of the joint and desired tasks in the ADLs. Some researchers have paid their concern on the powered bio inspired knee joint development and they have used many actuation methods such as electric motors, pneumatic artificial muscles and hydraulic linear actuators. Further beyond the hardware structure, some research work has moved for different control strategies to function the robot joint during the different human locomotion patterns. Some control strategies directly use the human desires as the input for the control algorithm to perform the desired joint output. The design attributes and control strategies of the 12 modern bio-inspired knee joint reviewed in this chapter is summarized in Table 1.

**TABLE 1:** General characteristic comparison of modern bio-inspired knee joints at research level.

Name and source	Bio-inspiration on	Actuation method	Knee joint characteristics			Desired tasks in ADLs	User tests
			Range of motion	Functional movements	Control strategy		
Condylar hinge joint by Etoundi et. al. (Hinson Marilyn, 1981)	Bone structure, ligament structure	N/A	0°–160°	Rolling and sliding	N/A	Walking, running	No
Knee joint for bipedal robot by Steel et al. (Kerrigan et al., 2000)	Bone structure, ligament structure	N/A	0°–160°	Rolling and sliding	N/A	Walking on rough terrain, running	No
				Dynamic control on contact surface			
				Function of patella			
Biomimicking design for mechanical knee by Russell et al. (Masahiro et al., 1990)	Bone structure, ligament structure	N/A	0°–120°	Rolling and sliding	N/A	Walking on rough terrain	No
				Function of patella		Walking stairs Sit-to-stand	
Tensegrity flexural joints by Jung et al. (Etoundi et al., 2011)	Bone structure, muscle and ligament structure	Electric motor	0°–110°	Rolling and sliding	N/A	Walking	No
Humanoid knee joint with patella by Asano et al. (Steele et al., 2017)	Bone structure, muscle and ligament structure	Linear actuators	0°–90°	Rolling and sliding	N/A	Walking on rough terrain	No
				Function of patella		Sit-to-stand	
				Screw-home mechanism		Yaw axis rotation	
Knee joint for exoskeleton by Pyo et al. (Russell et al., 2018)	Muscle and ligament structure	Linear actuators	0°–150°	Rotation on fixed axis function of patella	N/A	Walking, climbing slope during rehabilitation	No
Knee joint for power assist suit by Sakai et al. (Jung et al., 2018)	Muscle and ligament structure	Pneumatic artificial muscles	0°–160°	Rolling and sliding	N/A	Walking, climbing slope during rehabilitation	Yes
Knee joint for biped robots by Liu et al. (Asano et al., 2011)	Muscle and ligament structure	Pneumatic artificial muscles	0°–90°	Rotation in variable center of rotation	N/A	Walking on rough terrain, running	No
Articulated robotic leg by Li et al. (Pyo et al., 2010)	Muscle structure	Hydraulic linear actuator	45°–145°	Rotation on fixed axis	Active compliance control based on torque values	Walking on rough terrain, running, jumping	Yes
Knee joint for quadruped robot by Khan et al. (Liu et al., 2016)	Ligament structure	Hydraulic linear actuator	0°–180°	Rotation in variable center of rotation	N/A	Walking on rough terrain, running, jumping	Yes
Biomechatronic knee prosthesis by Torrealba et al. (Li et al., 2017)	Control architecture	Magnetorheological actuator	0°–85°	Rotation on fixed axis	Control via central pattern generator	Walking on rough terrain, running	Yes
Knee exoprosthetic device by Pagel et al. (Lawson et al., 2014)	Control architecture	Electric motor	0°–100°	Rotation in variable center of rotation	Control via NeurImp and HuMRAC	Walking on rough terrain	Yes

## 4 Proposed Bio-Inspired Knee Joint

The inspiration for the design of the exoskeleton knee joint is derived from the condylar knee joint mentioned in the literature (Masahiro et al., 1990; Etoundi et al., 2011; Etoundi et al., 2013b). Since the condylar knee joint design fulfills the necessary functions for prosthesis and robots, it was therefore adapted for the design of a lower limb exoskeleton structure. The main challenge in designing the exoskeleton knee joint was to use the cross four bar mechanism while preserving sufficient space for the user s knee. For the prosthetic joint, the condylar knee replaces a missing joint and therefore makes use of the entire space (Masahiro et al., 1990; Etoundi et al., 2011; Etoundi et al., 2013b) and has a more robust design. On the other hand, the exoskeleton knee joint supports the human joint by providing an accurate movement mimicking the human cruciate ligaments which are the kinematics driver for the rolling-sliding motion occurring between the femur and tibia.

### 4.1 Conceptual Design

The proposed knee joint for exoskeleton devices consists of 14 parts. Out of these, the two main parts are the tibia and the femur part. Both the parts have complementary curvature which allows them to roll while the joint is actuated. This rolling-sliding movement mimics the motion happening in the biological knee joint to an extent in order to conform to the kinematics of the human knee and also increase the life of the joint (Etoundi et al., 2013b). The tibia part also has a fixed extension to mimic the function of the patella and stop the rotation of the knee (Steele et al., 2017). The tibia and the femur part are designed to safely support the human legs through the usage of four velcro harnesses. A total of four links are used to connect the tibia part to the femur part in a similar way the actual anterior and posterior cruciate ligaments are connected. As observed for biological knees, the links representing the posterior cruciate ligaments are longer than the anterior cruciate ligaments.

### 4.2 Experiments and Results

**FIGURE 8:**
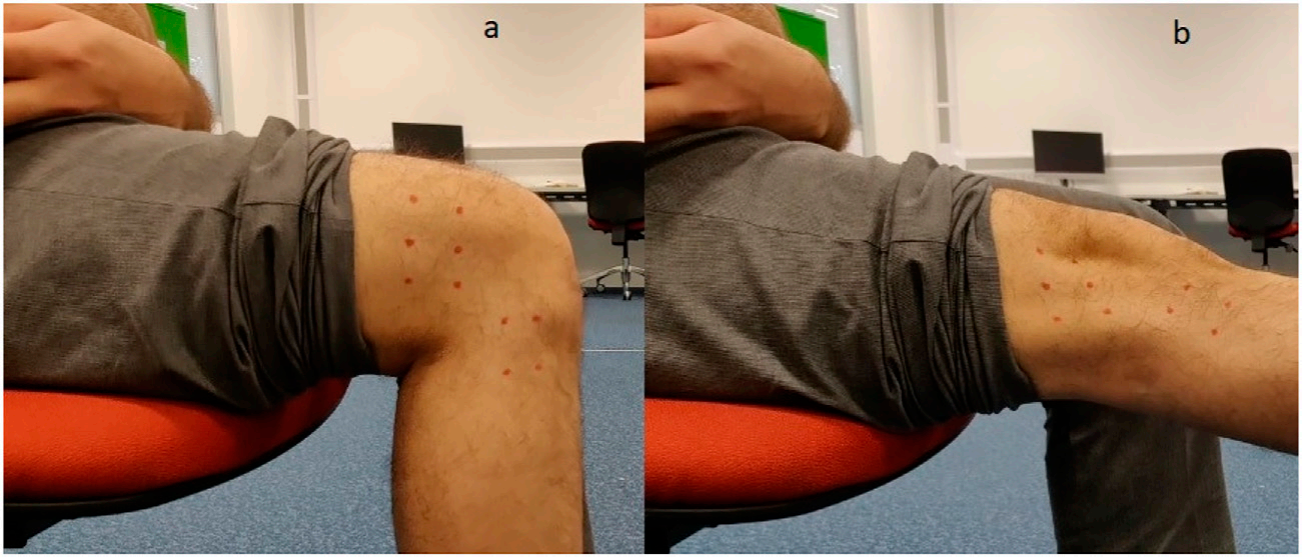
Motion profile test conducted on actual human knee: **(A)** knee in flexion, **(B)** knee in extension.

The fabricated knee joint was tested for conformity with range of motion of an actual knee joint and the sliding ratio between the femur and the tibia. The experiment consists of keeping the tibia fixed and moving the femur part of the fabricated joint while movements are being recorded by a high speed camera. Dark red spots are marked at four locations, two on the femur part and two on the tibia part to identify three key design criteria: i) tracking of the instantaneous centre of rotation; ii) finding the angle of rotation and iii) the sliding ratio. Tracking was done using an image processing code written in MATLAB which tracks the dark red spots and draws straight line in between them. The intersecting point of the lines is taken as the instantaneous centre of rotation of the femur and the tibia. In order to compare the data from the fabricated joint, baseline readings are taken in the same way from a human’s knee while they are seated in a steady position as seen in Figure 8. The points are marked around the knee to allow tracking of the leg’s movements. The initial position of the leg is at 90° flexion and the participant is asked to move the leg until it is straight and the knee is locked. Then, the participant moves back the leg to its initial position. This test method which was used to check the motion profile of the proposed exoskeleton knee joint is not explicitly based on existing experiment platforms. Reproducing the effects of established experiment platforms would require additional time. As an alternative, a simplified testing setup was used based on the working principle of bio-inspired test system for bionic above-knee prosthetic knees (Wang et al., 2013). The setup simulates a small section of the human gait when the knee starts extending (end of mid swing phase and late swing phase). No actuator was used for the experiment as the aim was to design a bio-inspired knee exoskeleton joint. The required motion was performed by fixing the tibia part on a stand and moving the femur part through a string as if pulled under the influence of a muscle. This was done to replicate the sliding motion occurring between the joints as much as possible. The motion profile of the joint was then compared to that of an actual human knee.

**FIGURE 9:**
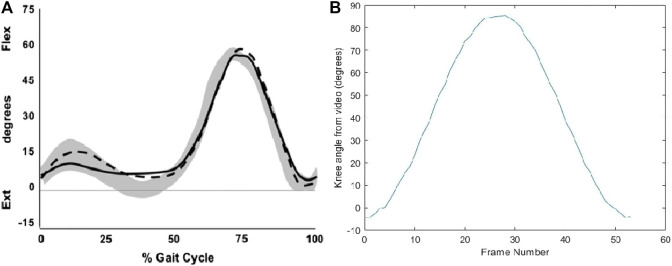
**(A)** Range of motion of knee during normal gait cycle (Martin et al., 2013), **(B)** Range of motion of fabricated knee joint exoskeleton.

**FIGURE 10:**
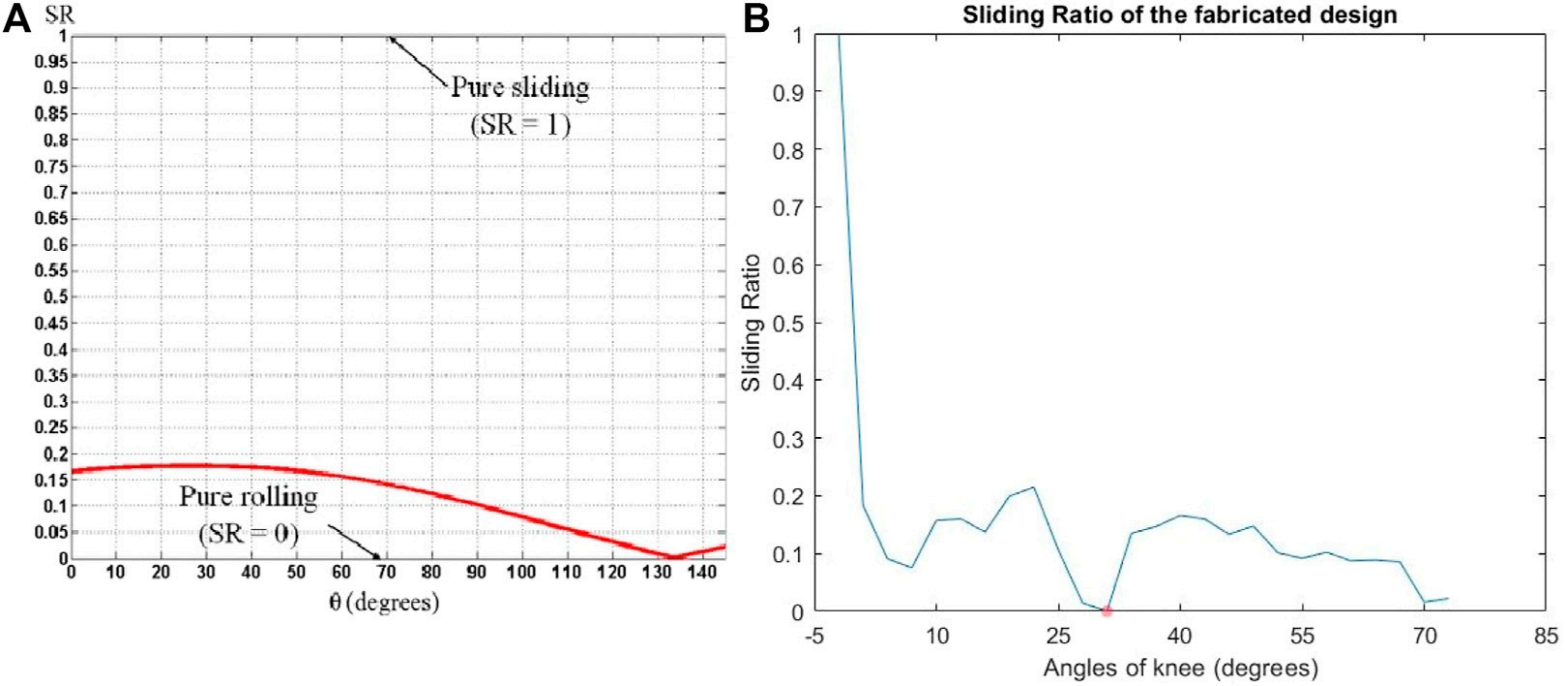
**(A)** Sliding ratio of prototype knee joint (Etoundi et al., 2013b), **(B)** Sliding ratio of fabricated knee joint exoskeleton.

The range of motion of the proposed knee joint in terms of the angle of the knee is shown in Figure 9. According to the experiment, the proposed knee joint covers the entire range of motion of the human knee needed for walking. The sliding ratio of the femur and tibia in the proposed design is shown in Figure 10. This graph gives information regarding the ratio of the sliding occurring against the rolling at the point of contact in the joint. This is necessary in order to predict the longevity of the joint. More sliding in the joint is associated with lesser life of the joint and an increased chance of mechanical failure. The sliding ratio of the presented joint is in line with the previous results of the authors for the research done on robotic limbs (Etoundi et al., 2013b).

The results show that the exoskeleton knee joint provides an acceptable performance, however, more assertive experiments need to be done in the form of real world tests such as mechanical stress test, range of motion test using an actuator and controller, and performance tests while the joint is being used by a person. The main aim of future research on this joint will be to incorporate a lightweight but high torque actuator and a suitable control algorithm to allow for autonomous or semi-autonomous operation of the joint. One important characteristic of the actual ligaments which would be incorporated is elasticity which allows the ligaments to stretch to about 15–20 percent more than the original length (Kennedy et al., 1977).

The design also needs to be refined in order to allow for easier wearing and removal while taking care that the user is not stressed at any point during the operation of the device.

## 5 Discussion and Future Direction

This paper presents a review of scientific works on design and development of bio-inspired knee joints used in prostheses, exoskeletons and biped robots in the past decade. It provides a groundwork for an ideal bio-inspired knee joint which mimics the human knee joint by eliminating all the limitations found within existing designs and combining all the possible strengths discussed in the review. In the analysis of anatomy and biomechanics of the human knee joint it is identified that a knee joint performs three key functions for stable, efficient, adaptive and compliant locomotion; rolling and sliding, patella movement and screw-home mechanism. There are many design approaches available in the existing the bio-inspired knee joints that achieve at least one key function found in human knee joint. Not only the hardware design, but also a good control architecture is needed to achieve human-like motions based on different control strategies and collection of data inputs. However, there is a considerable gap between an artificial knee joint and the human knee joint due to strict limitations such as compactness, weight, functionality, performance and aesthetic appearance. Four key aspects are considered in this review to benchmark the functional performance and compare the mechanical design and control architectures of existing bio-inspired knee joints; the bone structure, the muscle and ligament structure, the joint actuation method and the control strategy.

The bone structure (main links) of the joints in various scientific research are differentiated by the shape of the link components, material they are made of, number of DoF achieved, lay out for smooth relative motion between links and the patella arrangement. However, it is noted that mimicking the bone structure is extremely challenging for an ideal knee joint. For instance, the humanoid knee joint with patella by Asano et al. (Steele et al., 2017) mimics almost the same bone structure that is found in the human knee. Though it brings less power requirement for joint actuation, the range of motion of the knee has been reduced to 90° compared to range of motion found in condylar hinge joint by Etoundi et al. (Hinson Marilyn, 1981) which does not utilize the patella arrangement in the hardware design. The design approaches for mimicking the functions of muscles were reviewed based on the tendon or actuator attachment points on tibial and femoral links and compliance arrangements. In addition, ligament arrangement was also deeply analyzed, which is important in rolling and sliding function at knee flexion/extension. This sliding effect brings friction over the movements that makes a less efficient and durable joint. Though research on the knee joint for a bipedal robot by Steel et al. (Kerrigan et al., 2000) address those two limitations, the manufacturing and assembly process finds complex and controlling many actuators is very challenging for optimal knee movement. In this review, it is noted that there is less focus on function of the meniscus found in the human knee joint which disperses the load and impact forces while reducing friction at knee flexion/extension. Therefore the focus on menisci function in future designs may be beneficial for an ideal knee joint. Compliance and adaptation are also two key factors found within a human knee as it undergoes variable load and impact forces in locomotion. The actuators play a significant role on those factors There are some scientific works that has used pneumatic and hydraulic actuators to achieve adaptation and compliance along with other additional benefits. However, the power source for these actuation systems creates limitations in mobility, portability, user acceptance and aesthetic appearance of the device as whole. With regard to those limitations, electric actuators are favourable on the bio-inspired knee joint design. However, there is a serious need of a compliant and adaptive mechanism using electric actuation on the knee joint functionality with the concern of low power requirements.

Impedance control and control via CPG methods are the two common strategies inspired from biological motor control architecture for controlling the knee joint. The control inputs for these strategies are gathered from sensors which detect motion and forces. Control architecture is built based on a mathematical model and a neural network. So this brings involuntary movement of the knee joint that supports human-like motion during locomotion. This restricts the voluntary control of the knee that a healthy human can perform. There is limited research on voluntary control with the use of physiological control inputs such as electrocardiography (EMG), mechanomyography (MMG) and electroencephalography (EEG) signals. By combining the existing control architectures with physiological control signals, an ideal knee joint can be developed for prostheses and exoskeletons.

In terms of the proposed exoskeleton design, the knee joint has been made to closely mimic the function of the biological knee joint but distilling the underlying principle of the knee joint and incorporating it into the structure of the proposed design. The various lengths of the tibia, femur, and the connecting cruciate ligaments have been taken into consideration while designing the mechanism of the proposed knee joint for exoskeletons. The motion profile of the design was repeatedly simulated to closely match that of the biological human knee before deciding on the final design. This has made it possible for the proposed design to almost match the biological knee in terms of range of motion while walking and the sliding ratio.

The next part of the research will be to incorporate semi-active spring based characteristics into the links of the proposed design which are analogous to the cruciate ligaments in the biological knee. Further research would look into dealing with the problem of finding or building a suitable actuator for the exoskeleton knee joint without adding unnecessary bulk and weight and the accompanying control architecture. Another aspect to consider is the testing of these developed joints. In order to properly test the joints, it is necessary to use a test rig which can repeatedly stress the joints according the motion profile of the human knee and subsequently measure the resulting torque on the knee. Research on such a test rig is already under progress and would prove to be a valuable addition to the entire bio-inspired design ecosystem.

## 6 Conclusion

This paper briefly review the hardware designs and control strategies of modern bio-inspired knee joints found in the research field of rehabilitation, power-assist suits and mobile robots. From the comprehensive review conducted, the key factors such as size, weight, appearance, durability, performance and functional capabilities are identified as the major concerns for design and development of robotic knee joints. In addition there is a serious need for testings and evaluations of the bio-inspired knee joint by the means of functional capabilities, efficiency, user acceptance, ergonomics, reliability, aesthetic appearance and the cost and ease of manufacturing. However, it is admissible from the review that the bio-inspiration may build the path to achieve the most desired concerns for robotic knee joint towards the safe human-like movement. The bio-inspiration can be brought to the knee joint development by mimicking the four main aspects found in the human knee namely bone structure, ligament structure, muscle structure and neural control structure. Based on these findings, a conceptual knee joint design is proposed in this paper for a lower limb exoskeleton. In the joint design, biological human knee structure is taken as the base where the curved or condylar shaped joint is proposed. The experimental results have shown that the proposed structural design has enabled the joint to function with high conformity, strength and stiffness. In addition, the linkage mechanism with changing center of rotation which is embedded in the proposed design, has been able to achieve the optimal mechanical advantage and desired sliding ratio of the femur and tibia during the joint movement. From the experimental results, it is also noticed that the conceptual design has the functionality of locking in the upright position which is the most essential in standing posture. However, there are some design alterations to be done to actuate the conceptual knee joint. As the future work, an actuation system will be designed and implemented to this knee joint with a hybrid control architecture built with combination of impedance control and bio-signal based control.

## Data Availability

The original contributions presented in the study are included in the article/supplementary material, further inquiries can be directed to the corresponding author.

## Footnotes

1 http://www.cyberdyne.jp/ (Accessed July 26, 2021).

2 https://innophys.jp/ (Accessed July 26, 2021).
